# *Mycoplasma pneumoniae‐*associated encephalitis complicated by cerebral salt wasting syndrome

**DOI:** 10.1002/ccr3.1192

**Published:** 2017-09-26

**Authors:** Ya‐Lan Lin, Kun‐Long Hung, Chiao‐Wei Lo

**Affiliations:** ^1^ Department of Pediatrics Cathay General Hospital Taipei Taiwan; ^2^ School of Medicine Fu Jen Catholic University New Taipei Taiwan

**Keywords:** Cerebral salt wasting syndrome, encephalitis, hyponatremia, *Mycoplasma pneumoniae*

## Abstract

Cerebral salt wasting syndrome can occur in children with encephalitis. Clinicians should be aware of hyponatremia in patients who develop polyuria with the signs of dehydration and deteriorated consciousness. Furthermore, patients who present with status epilepticus or who are suspected to have high intracranial pressure may have an increased risk of cerebral salt wasting syndrome.

## Introduction

Hyponatremia is a common electrolyte imbalance that occurs in children. It can be attributed to many causes such as dehydration, the increased release of antidiuretic hormone, and the administration of hypotonic intravenous solutions 1. Hyponatremia is commonly found in patients with central nervous system diseases, particularly after subarachnoid hemorrhage 2.

Cerebral salt wasting syndrome (CSWS) is defined as the renal loss of sodium caused by intracranial disorders, leading to hyponatremia and a decrease in extracellular fluid volume 3. In children, the causes of CSWS range from intracranial surgery, meningoencephalitis, head injury, intracranial bleeding, and hydrocephalus. In patients with meningoencephalitis, tuberculous meningitis is the most common disease 4. Here, we report a case of an 8‐year‐old girl who developed *Mycoplasma pneumoniae*‐associated encephalitis with the subsequent insult of CSWS.

## Case Report

An 8‐year‐old girl presented to the emergency department with reduced consciousness and an 8‐day history of high fever. Before admission, she had some vomiting episodes and epigastric pain combined with mild cough. On arrival, her pulse rate was 112 per minute, respiratory rate was 19 per minute, blood pressure was 93/62 mm Hg, and temperature was 36°C. Physical examination results were normal.

**Figure 1:**
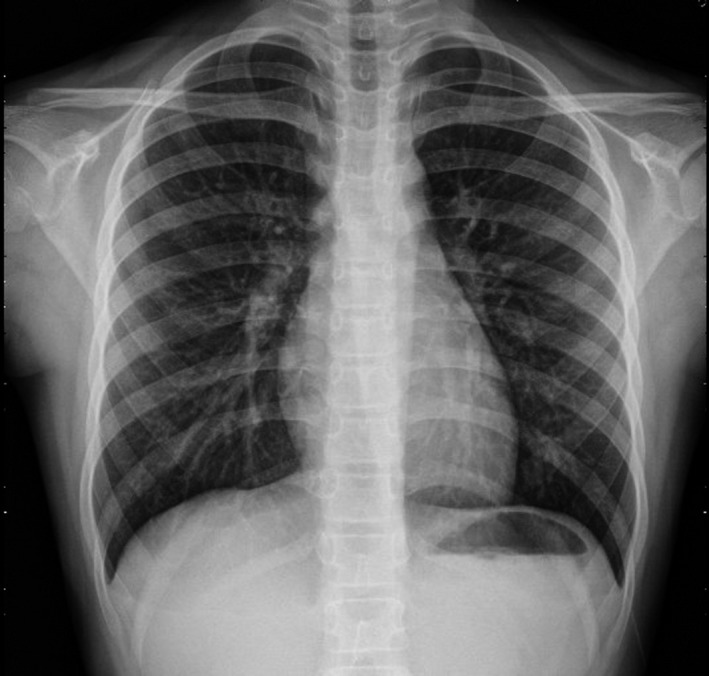
Chest radiography of the patient on first day of admission. Slight increase observed in markings in bilateral lungs.

On neurological examination, she was drowsy and disoriented (Glasgow coma scale [GCS]: 14 of 15; eye opening: 4, motor response: 6, and verbal: 4). Memory was impaired, and visual hallucination and unsteady gait were noted. Deep tendon reflexes were normal, and plantar responses were flexor. The initial blood test showed acute respiratory acidosis without compensation and mildly elevated liver enzyme levels. The results of other routine biochemical, hematology, and electrolyte tests were normal, and sodium, blood urea nitrogen, and creatinine levels were 135 mEq/L, 11 mg/dL, and 0.49 mg/dL, respectively. Chest radiography revealed a slight increase in bilateral lung markings (Fig. 1).

Because of decreased consciousness, brain computed tomography was performed and revealed unremarkable findings. After the patient was admitted to the pediatric intensive care unit, we performed lumbar puncture. Cerebrospinal fluid (CSF) analysis revealed normal protein (26 mg/dL) and glucose (82 mg/dL) levels and a normal white blood cell count (5/*μ*L), with lymphocyte predominance. Approximately 12 h after admission, she suddenly developed complex partial seizure with secondary generalization. Intravenous phenytoin was administered, and acyclovir was administered because viral encephalitis was initially suspected. On the third day of admission, the patient's febrile condition persisted; the frequency of seizures increased, and the seizures were complicated by several episodes of auditory and visual hallucinations. The patient received as the second antiepileptic medication. Brain magnetic resonance imaging results were normal. Serological studies revealed that herpes simplex virus (HSV) I IgM, HSV II IgM, cytomegalovirus IgM, and Epstein–Barr virus IgM were all negative, but *M. pneumoniae* IgM was positive (3.44). Azithromycin was prescribed for 5 days. Blood and CSF cultures were sterile. Electroencephalography (EEG) showed diffuse slow waves on background activity, rhythmic discharge in parietal–temporal regions for 20″–30″, and focal paroxysmal spikes in the left parietal area.

On day 4, seizures occurred almost hourly, and the pattern had changed to the generalized myoclonic type.

**Figure 2:**
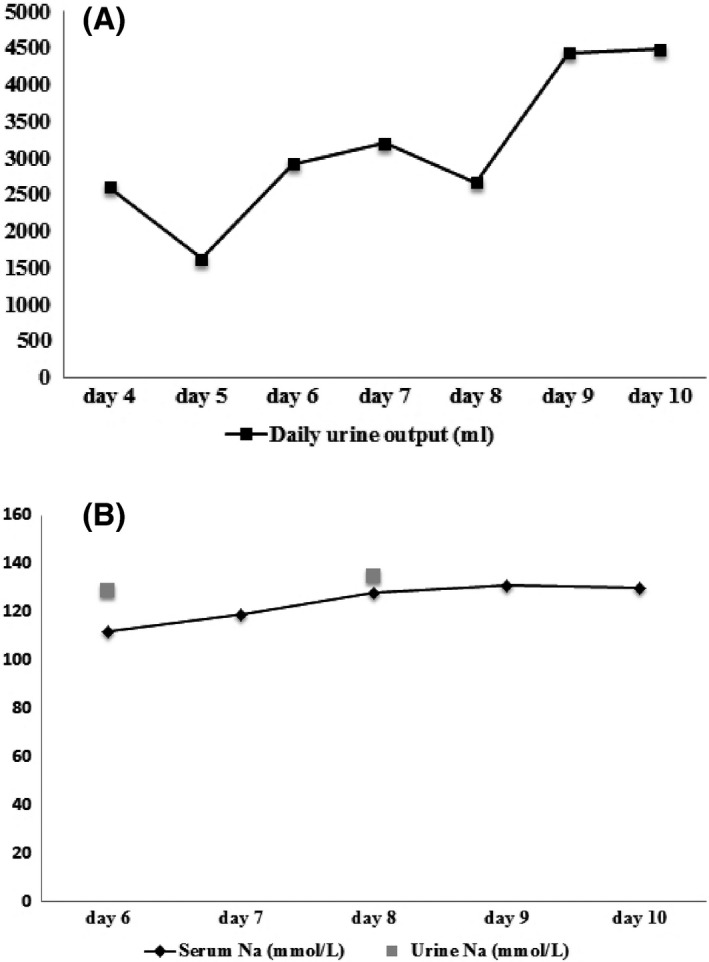
(A) Daily urine output of the patient. Polyuria was observed from the sixth to tenth day of admission. (B) Serum sodium and urine sodium levels after admission. The sodium level in urine was greater than that in serum during two urine analyzes.

For further seizure control, oxcarbazepine and valproate were prescribed in addition to levetiracetam and phenytoin. However, deteriorated consciousness (GCS: E2V2M5) combined with polyuria was noted. The patient's daily water intake was 1324 mL, and urine output was 2606 mL (Fig. 2A). In addition, the signs of dehydration, including decreased skin turgor, tachycardia, and body weight loss (−0.6 kg), were detected. Despite adequate fluid replacement, a negative fluid balance was still noted. On day 6, laboratory findings revealed a serum sodium level of 112 mmol/L and serum osmolarity of 227 mosmol/kg. The patient's urinary sodium level was 129 mmol/L (Fig. 2B), urinary osmolarity was 437 mosmol/kg, and fractional excretion of sodium was 2.76 × 10^−2^. Hypotonic hyponatremia was attributed to renal loss of sodium. The patient was hydrated with hypertonic saline. On the following day, her circulating sodium level was 124 mmol/L and her urine output slowly decreased. She was afebrile and regained consciousness. On day 9, she was seizure free. Hyponatremia and dehydration also gradually improved. On day 21, a follow‐up EEG revealed bilateral periodic lateralized epileptiform during sleep recording.

## Discussion

Many review articles and studies have discussed how to distinguish CSWS from syndrome of inappropriate antidiuretic hormone secretion (SIADH). In this case report, the patient was diagnosed as having CSWS because of the following findings: (i) polyuria was noted during the clinical course; (ii) the patient had hypotonic hyponatremia; (iii) hypovolemia was diagnosed based on the signs of dehydration; and (iv) renal loss of sodium and a negative sodium balance were noted (Table 1). Jiménez et al. 5 described the following selection criteria for CSWS: evidence of hyponatremia (serum sodium <130 mEq/L), polyuria, elevated urine sodium (>120 mEq/L), and volume depletion. Our patient fulfilled all criteria.

**Table 1:** Differential diagnosis 3, 4. Comparison between cerebral salt wasting syndrome (CSWS) and syndrome of inappropriate antidiuretic hormone secretion (SIADH). The plasma volume, fluid balance, and urine output are the key factors for differentiating between CSWS and SIADH

	Cerebral salt wasting syndrome	Syndrome of inappropriate antidiuretic hormone secretion
Plasma volume	↓	↑
Fluid balance	Negative	↑ or normal
Natriuresis	Marked ↑↑	↑ but not high
Salt balance	Negative	Variable
Urine output	Polyuria	Decreased or normal
Uric acid	↓	↓
Treatment	Response to saline and fludrocortisone	Response to fluid restriction and furosemide

↓, decrease; ↑, increase.

Notably, the fractional excretion of sodium also increases in patients with SIADH, primarily because of vasopressin stimulation and secondarily because of overfilling of the plasma volume through a homeostatic mechanism 6.

Therefore, the definitive characteristic that distinguishes CSW from SIADH is volume status. However, it is difficult to evaluate volume status in children. Ohand Shin 6 have shown that the uric acid level may be helpful for distinguishing CSW from SIADH. The initial serum uric acid level is low in CSWS and SIADH. Hypouricemia and the increased fractional excretion of uric acid improve after the correction of hyponatremia in patients with SIADH, but not in those with CSWS. Therefore, serial studies rather than one‐time measurement of the uric acid level may be needed to differentiate CSWS from SIADH.

To date, the mechanism of CSWS had not been clarified. Some studies 2, 3, 7 have found that CSWS is associated with increased atrial natriuretic peptide (ANP) and brain natriuretic peptide (BNP) levels. Previous studies have also reported a direct correlation of ANP and BNP levels with intracranial pressure (ICP), suggesting that patients develop renal salt wasting as a protective measure 3, 7.

Our patient had status epilepticus; therefore, increased intracranial pressure was suspected. Çelik et al. also reported two cases of status epilepticus with CSWS 8. A previous study suggested that the increases in BNP and ANP levels are triggered by epileptic stimulation 9. However, not every patient with CSWS has an increased BNP level; therefore, the causes may differ depending on the mechanism of CSWS 10.

In addition, notably, the CSF and blood cultures of our patient were sterile, and only serum *M. pneumoniae* IgM was positive. Although the patient only had mild respiratory symptoms before admission, it was feasible that acute encephalitis was caused by *M. pneumoniae*, as described by Al‐Zaidy et al., that *M. pneumoniae* could have directly or indirectly infected the central nervous system 11. In addition, our patient had a prolonged prodrome (>7 days), which may have been immunologically mediated. We could detect *M. pneumoniae* in the respiratory tract, but not in the CSF.

Tuberculous meningitis is the most common infection in patients with meningoencephalitis 4. The risk of increased ICP is higher in tuberculous meningitis than in other infections observed in these patients. Moreover, hydrocephalus is a severe complication of tuberculous meningitis and can result in highly increased ICP. Huang et al. reported a case of tuberculous meningitis with prolonged CSWS, which improved after hydrocephalus was resolved through placement of a ventriculoperitoneal shunt 12. Çelik et al. also emphasized the importance of relieving hydrocephalus in patients with tuberculous meningitis because it was correlated with an improvement of CSWS 13. Drawing on previous reports and studies, we hypothesize that the level of increased ICP is crucial, rather than infectious pathogens inducing CSWS.

## Conclusion

Cerebral salt wasting syndrome is a rare cause of hyponatremia in children; however, using careful physical examination and laboratory tests, CSWS can be diagnosed in patients and effective management for hyponatremia is possible. Our patient exhibited mycoplasma pneumoniae‐associated encephalitis, which was complicated by status epilepticus, resulting in CSWS. Previous studies and reports have suggested that status epilepticus and increased ICP are correlated with CSWS; both conditions are probably related. Alternatively, these conditions may cause CSWS through different mechanisms. We expect that future studies will elucidate the mechanisms underlying CSWS.

## Authorship

YLL: involved in the literature search, writing up initial drafts, later revisions and meetings between authors. KLH: involved in coordinating correspondences, assistance in subsequent edits and finalizing manuscript. CWL: involved in the literature search and assistance in subsequent edits.

## Conflict of Interest

None declared.
